# Diverse mobile genetic elements shaped the evolution of *Streptomyces* virulence

**DOI:** 10.1099/mgen.0.001127

**Published:** 2023-11-06

**Authors:** Alexandra J. Weisberg, Emma Pearce, Charles G. Kramer, Jeff H. Chang, Christopher R. Clarke

**Affiliations:** ^1^​ Department of Botany and Plant Pathology, Oregon State University, Corvallis, OR 97331, USA; ^2^​ USDA Agricultural Research Service, USDA Agricultural Research Service, Genetic Improvement for Fruits and Vegetables Lab, Beltsville, MD, USA

**Keywords:** evolution, mobile genetic elements, integrative conjugative elements, *Streptomyces*, phytopathogen

## Abstract

Mobile genetic elements can innovate bacteria with new traits. In plant pathogenic *Streptomyces,* frequent and recent acquisition of integrative and conjugative or mobilizable genetic elements is predicted to lead to the emergence of new lineages that gained the capacity to synthesize Thaxtomin, a phytotoxin neccesary for induction of common scab disease on tuber and root crops. Here, we identified components of the *Streptomyces *-potato pathosystem implicated in virulence and investigated them as a nested and interacting system to reevaluate evolutionary models. We sequenced and analysed genomes of 166 strains isolated from over six decades of sampling primarily from field-grown potatoes. Virulence genes were associated to multiple subtypes of genetic elements differing in mechanisms of transmission and evolutionary histories. Evidence is consistent with few ancient acquisition events followed by recurrent loss or swaps of elements carrying Thaxtomin A-associated genes. Subtypes of another genetic element implicated in virulence are more distributed across *Streptomyces *. However, neither the subtype classification of genetic elements containing virulence genes nor taxonomic identity was predictive of pathogenicity on potato. Last, findings suggested that phytopathogenic strains are generally endemic to potato fields and some lineages were established by historical spread and further dispersed by few recent transmission events. Results from a hierarchical and system-wide characterization refine our understanding by revealing multiple mechanisms that gene and bacterial dispersion have had on shaping the evolution of a Gram-positive pathogen in agricultural settings.

## Data Summary

Short reads and genome assemblies of strains sequenced in this study have been deposited in NCBI as BioProject PRJNA934919, and accession numbers are listed in Table S1, available in the online version of this article. Strains sequenced in this study are available from CRC upon request. The authors confirm all supporting data, code and protocols have been provided within the article or through supplementary data files.

**Impact Statement**

*
Streptomyces* is a diverse genus of Actinomycetota that includes more than ten species that can cause common scab, a devastating disease of potato and other tuber and root crops. The primary virulence determinants for common scab are found in mobile genetic elements (MGEs). Through analysis of 166 newly sequenced *Streptomyces* genomes, this work characterizes the species diversity of phytopathogenic *Streptomyces* and the interactions between different types and subtypes of MGEs implicated in plant pathogenicity through robust classification and categorization of MGEs. Analyses revealed that interactions between different mobile elements generated new genetic diversity and combinations of virulence genes within strains. Second, genomic epidemiological analyses revealed that transmission of phytopathogenic *Streptomyces* within North America was likely prevalent in the past but rare within the last century. Using this improved and deeper characterization of *Streptomyces* and its mobile genetic elements, this work provides a detailed evolutionary model that integrates multiple mechanisms that have shaped phytopathogenicity within the genus to refine our understanding on the evolution of *Streptomyces* phytopathogenicity.

## Introduction

Mobile and mobilizable genetic elements (MGEs) are associated with the rapid evolution of bacteria as well as the emergence of new species and lineages [1–3]. Horizontal acquisition (horizontal gene transfer; HGT) of genes can add new traits whereas integration of elements can disrupt or alter gene function [4]. Importantly, MGEs are extremely plastic and promote rapid divergence by swapping genes within and across different elements and with chromosomes. As such, characterization of traits mobilizable by MGEs requires framing the study of components as a system of nested and interacting parts [5]. These components may include genes underlying a trait, the elements that vector the genes, the bacteria that host the elements, and in the case of symbiotic or pathogenic microbes, the host. Use of such an approach has revealed multiple mechanisms and the degree to which MGEs contribute to emergence of bacterial lineages in agricultural and clinical settings [6–10].

MGEs include molecules, such as plasmids, integrative and conjugative elements (ICEs), insertion sequences and transposons. Plasmids replicate autonomously while ICEs integrate into chromosomes and replicate passively along with them [11]. Conjugative plasmids and ICEs encode a type IV secretion system (T4SS) and autonomously mediate their transfer to recipient cells [1]. Conversely, mobilizable plasmids and integrative and mobilizable elements (IMEs) lack a T4SS and their transfer is dependent on functions encoded by other elements [12].

*Streptomyces* is a genotypically and phenotypically diverse genus of Actinomycetota bacteria found ubiquitously in soil ecosystems. The *Streptomyces* life cycle includes phases of both filamentous mycelial growth and production of aerial hyphae that produce spores. Members of the genus produce diverse secondary metabolites and are intensively studied because of their value [13]. Many *Streptomyces* species are harmless saprophytes found in the plant rhizosphere, and some promote plant growth [14]. However, more than ten species include plant pathogens that cause common scab disease. This manifests as superficial, raised or pitted lesions, on potato and other tuber and root crops [15, 16]. Most of the known pathogenic species are present in North America [17, 18].

Disease by *Streptomyces* is primarily associated with the *txt* genes, which are necessary for the synthesis of phytotoxin Thaxtomin A [19–23]. The most common arrangement of *txt* genes is typified in *S. scabiei *. The genes are present in toxigenic region 1 (TR1, an IME) and tandemly integrated along with TR2 (an ICE) in *att* sites located in the 3′ end of *aviX1* [24]. TR2 lacks genes involved directly in virulence but encodes the capacity to transmit itself as well as TR1. However, *Streptomyces* strains have been identified with only TR1 or lacking both [25]. Two other virulence-associated genes, *tomA* and *nec1*, are often present in an ~100 kb long colonization region (CR) integrated in the 3′ end of *bacA* [23]. In most species characterized to date, CR and TR1 are physically separated from each other [25]. Conversely, a 660 kb region in *S. turgidiscabies* strain Car8 carries both CR and *txt* genes in separate MGEs that are tandemly integrated in *bacA* [19]. In this arrangement, CR and *txt* regions are modular, separated by an internal *att* site and can be independently excised [26]. Last are the pathogenic *S. ipomoeae* species, which produces Thaxtomin C, a less-modified derivative that is essential for virulence on sweet potato [27], and other strains that do not produce Thaxtomin A and cause netted scab disease [28, 29].

The *txt* genes were hypothesized to be necessary and sufficient for *Streptomyces* to cause common scab disease. Strains originally non-pathogenic gained the ability to cause disease when they received the large 660 kb co-integrated MGEs of *S. turgidiscabies* strain Car8 [20, 25, 30–32]. However, surprisingly, multiple non-pathogenic strains have been identified that natively maintain an intact TR1 IME [33, 34]. One key difference is that *tomA, nec1* and *fas*, implicated in virulence, are present in the tandem ICE of strain Car8, but absent from TR1 [35–38]. But this is not likely explanatory because presence/absence of these virulence-associated genes is not predictive of strain pathogenicity [17, 18].

Horizontal acquisition of *txt-*carrying ICEs and IMEs has been implicated as the major driver in shaping the evolution of phytopathogenicity within the genus. Support has been largely based on conjugation experiments in the laboratory and incongruencies between *txt* gene and *Streptomyces* tree topologies [24]. It was originally suggested that HGT of TR1-TR2 occurred among *S. scabiei, S. acidiscabies* and *S. turgidiscabies* species [39]. A revision suggested that HGT additionally involved *S. caniscabiei *, represented by strain 96–12 [24, 40]. In contrast, the evolution of *S. ipomoeae* was predicted to be different, as its *txt* genes were inferred to trace back to a common ancestor shared with *S. scabiei* but having had diversified since separation of species [39]. Because pathogenic strains cluster closely with non-pathogenic strains, HGT was associated with the recent emergence of multiple pathogenic species [24, 25]. However, sample selection and high homology of *txt* genes make it difficult to distinguish between HGT and gene loss within a lineage. The contributions of HGT, particularly recent events, and vertical inheritance in shaping the evolution of plant pathogenic *Streptomyces* remains unclear.

Here, we reevaluated models that predicted the evolution of plant pathogenic *Streptomyces *. To this end, we sequenced genomes from a population of *Streptomyces* strains and constructed a deep genus phylogeny. We robustly classified types and subtypes of virulence-associated MGEs. The evolutionary histories of virulence genes and MGEs were inferred separately, and findings were framed relative to the system of components that influence phytopathogenicity [9, 41]. We provide a detailed model that predicts the contributions of horizontal and vertical inheritance of MGEs, their losses, as well as bacterial spread on the evolution of Gram-positive *Streptomyces* pathogens in agricultural settings.

## Methods

### Bacterial growth and genome sequencing

Strains of *Streptomyces* were cultured on 1.8 % agar plates of yeast malt extract (YME) or Oat Bran media (Table S1; [42]). Spores were harvested by resuspending in double-distilled, autoclaved water (ddH_2_O). Spore suspensions were added to 30 ml of YME and shaken at 200 r.p.m., 28 °C for 24–48 h. Liquid cultures were centrifuged at 4000 r.p.m. for 60 min to pellet bacterial mycelial mass. Pellets were resuspended in approximately 2 ml of ddH_2_O and treated with 200 µl of 10 mg ml^−1^ lysozyme in 50 mM EDTA for 60 min at 37 °C. Genomic DNA was extracted from cell suspensions by following manufacturer’s protocol (Wizard DNA Purification kit; Promega, Madison, WI, USA).

DNA samples were sequenced either by Genewiz LLC (South Plainfield NJ, USA) or the Center for Quantitative Life Sciences (CQLS; Oregon State University, Corvallis, OR, USA). Sequencing (150 bp paired-end reads) by Genewiz and CQLS was performed on an Illumina HiSeq 3000. For long-read sequencing, strains were sequenced on a Flongle flowcell (R9.4.1) on a Mk1b MinION sequencer controlled by a MinIT coprocessor. Samples were prepared using a Ligation Sequencing Kit (SQK-LSK110) prior to sequencing.

### Genomic analyses

Previously described methods were used to assemble, annotate and analyse genome sequences [41]. FastANI v. 1.1 with the default options was used to calculate pairwise average nucleotide identity (ANI) and based on a threshold ≥95 %, operationally classify strains into species-level groups [43]. pirate v. 1.0.4 with the options ‘--pan-opt '--diamond' --align -z 0 --para-off’ was used to cluster genes into orthologous groups [44]. Publicly available *Streptomyces* genome sequences were downloaded from the NCBI RefSeq database on 27 June 2019. wgsim v. 0.3.1-r13 with the options ‘-N 1000000–1 150–2 150 r 0 R 0 X 0 -e 0’ was used to simulate paired sequencing reads for publicly available assemblies without available reads [45].

### Construction of phylogenetic trees

The 540 single-copy (95 %) core genes of *Streptomyces *, as determined by pirate orthologue clustering analysis, were used to construct a core-genome phylogeny based on nucleotide sequence. mafft v. 7.487 with the default options was used to generate multiple sequence alignments for each core gene, as well as for all other sequence datasets [46]. iq-tree v. 1.6.12 with the options ‘--ufboot 1000 --alrt 1000 m GTR+I+G’ and the individual core gene alignments was used to generate a core-genome phylogeny [47]. iq-tree v. 1.6.12 with the options ‘-bb 1000 -alrt 1000’ was used to generate phylogenies for all other sequence datasets [47]. The R package ggtree was used to plot phylogenies [48]. The R packages phytools and ggtree were used to generate cophylo plots [48, 49]. For gene presence/absence matrices, hierarchical clustering was based on ward.D2 clustering of binary distances.

### Analysis of ICE/IME regions

Previously described methods were used to scaffold and identify genetic elements in draft genome sequences [41]. blastn v. 2.13.0+searches were used to identify putative border sequences in scaffolded genome assemblies [50]. The following border sequences were used as queries: TAGACGTTGAAGCGGAAC (*aviX1*-associated islands; TR1.A, TR2), ACCTCCGCCGCCAGGTCCAGTCGTTCAAGGGGAGGC (CR.B), GCTCGGTTT (CR.C), and GGGCCGCTGTGGCCGGCTGCACCATGTACTCACTG (TR1.C; novel IME). Fragments of *bacA* from strains 87.22 and Car8 were used as queries for *bacA*-associated islands. ProgressiveMauve with the default options was used to compare complete genome assemblies and identify putative boundary regions of novel ICEs/IMEs [51].

Gene presence/absence matrices for each MGE type and their subtypes were inferred from the pirate orthologue clustering analysis. To normalize between different gene annotation pipelines, TR1 islands from genome sequences retrieved from NCBI were re-annotated using prokka v. 1.14.6 with the default options, and TR1 elements were re-analysed using pirate to place their genes into orthologous groups [52]. The R package heatmap.plus or the ggtree function gheatmap were used to generate gene presence/absence heatmaps [48, 53]. Sourmash v. 2.0.0a11 with the options ‘compute -scaled 100’ and ‘compare -k 21’ was used to estimate a Jaccard Index based on *k*-mer signatures [54]. These values were used to build a graph with MGEs as nodes, and edges linking nodes with a Jaccard Index≥0.1. Cytoscape v. 3.9.0 was used to visualize these graphs [55]. The last v. 1066 programme lastal with the option ‘-f BlastTab+’ was used to align sequences of MGEs, and alignments were visualized using the BioPython module GenomeDiagram v. 1.72 [56, 57].

SNP analysis of TR1 and CR.A islands was done using regions of strain 87.22 as references. bwa v. 0.7.17-r1188 with the options ‘mem -M’ was used to align paired reads from each strain to its own scaffolded genome assembly [58]. Samtools v. 1.9 was used to extract reads mapping to the TR1 or CR region of each strain [59]. bwa v. 0.7.17-r1188 with the options ‘mem -M’ was used to align extracted sequencing reads to the reference sequence [58]. Picard tools v. 2.18.27 was used to add read groups, mark duplicates, sort and index bam alignments [60]. The gatk v. 3.7 programme HaplotypeCaller with the options ‘-ERC GVCF -ploidy 1’ was used to call SNPs for each sample [61]. The gatk v. 3.7 programme GenotypeGVCFs was used to merge and reconcile individual SNP calls. The gatk v. 3.7 programmes SelectVariants with the option ‘-selectType SNP’ and ‘--excludeFiltered’ and VariantFiltration with the options ‘--filterExpression ‘QD <2.0’ --filterName ‘QD2’ --filterExpression ‘SOR>3.0’ --filterName ‘SOR3’ --filterExpression ‘QUAL<30.0’ --filterName ‘QUAL30’ --filterExpression ‘FS >60.0’ --filterName ‘FS60’ --filterExpression ‘MQ <40.0’ --filterName ‘MQ40’ --filterExpression ‘MQRankSum <−12.5’ --filterName ‘MQRankSum-4’ --filterExpression ‘ReadPosRankSum <−8.0’ --filterName ‘ReadPosRankSum-8’ were used to filter out structural variant calls and filter SNPs for quality, respectively. The R package poppr was used to classify islands into genotypes and plot minimum spanning networks [62].

### Whole-genome SNP calling

Each ANI group was analysed independently with the most completely assembled genome sequence from each group used as references. bwa v. 0.7.17-r1188 with the options ‘mem -M’ was used to align sequencing reads to the reference genome sequence [58]. Picard tools v. 2.18.27 was used to add read groups, mark duplicates, sort, and index bam alignments [60]. Graphtyper v. 2.7.3 with the ‘genotype’ subcommand and the default options was used to call SNPs for each reference contig [63]. The graphtyper subcommand ‘vcf_concatenate’ was used to merge results. The gatk v. 3.7 programme VariantFiltration with the options ‘-G_filter ‘isHet==1’ -G_filterName ‘isHetFilter’ –setFilteredGtToNocall’ was used to filter heterozygous SNP calls [61]. The programme vcffilter with the options ‘-f ‘ABHet <0.0 | ABHet >0.33’ -f ‘ABHom <0.0 | ABHom >0.97’ -f ‘MaxAASR >0.4’ -f ‘MQ >30’’ was used to filter SNP calls for quality [64]. The gatk v. 3.7 programme VariantFiltration was used to mask regions of the reference genome corresponding to PAI and CR islands [61]. The R package poppr was used to classify strains into genotypes and plot minimum spanning networks [62].

### Plant pathogenicity assays

Virulence to radish seedlings was quantified as previously described [33]. Briefly, Radish *cv*. Champion (Ferry Morse) seeds were sterilized and sowed into 12-well plates with 0.4 % water agar. A total of 30 ml of YME was inoculated to a final concentration of 2000 *Streptomyces* spores/µl, grown at 28 °C for 3 days, mycelial mass pelleted via centrifugation, and then mixed 1 : 1 with ddH2O. Then, 30 µl of the *Streptomyces* mycelial suspensions were added to the germinated radish seedlings. The plates were covered and transferred to a plant growth chamber (Percival LED41L2) with 12 h life cycle and 29 °C for 9 days before measuring radish hypocotyl length, root length and cotyledon cross section. Six replicate radish plants were tested for each strain in each of two independent experiments with all data combined for analyses. Kruskal–Wallis non-parametric tests were performed in R to determine if significant differences in radish stunting exist across species, CR subtypes, TR subtypes or strains. Boxplots were generated in R, using ggplot2 with outliers marked as black dots.

Virulence assays on potato were done as previously described [33]. Potatoes were grown in 6 inch pots with 1 : 1 sand:potting mix (Metromix Sungro 830) in a greenhouse. *Streptomyes* mycelial liquid cultures were inoculated into sterile vermiculite and incubated at 28 °C for 2 weeks. The amount of c.f.u. g^−1^ of vermiculite was then quantified through dilution plating, and vermiculite was mixed into the sand:potting soil mixture to a final concentration of 2×10^7^ c.f.u./pot in the pots with the seed tubers. All daughter tubers were harvested after 12 weeks and scored for disease severity from each of three replicate pots (approximately six tubers per pot). Strains were tested in sets, with each set including negative mock treatments and positive treatments with inoculations of *S. scabiei* ME01-11h, one of the most aggressive strains in our collection [65]. For each strain treatment, disease scores were normalized to the disease score of positive control ME01-11h-infected tubers averaged across all sets.

## Results

### Diverse species of *Streptomyces* are associated with potatoes grown in North America

**Fig. 1.**
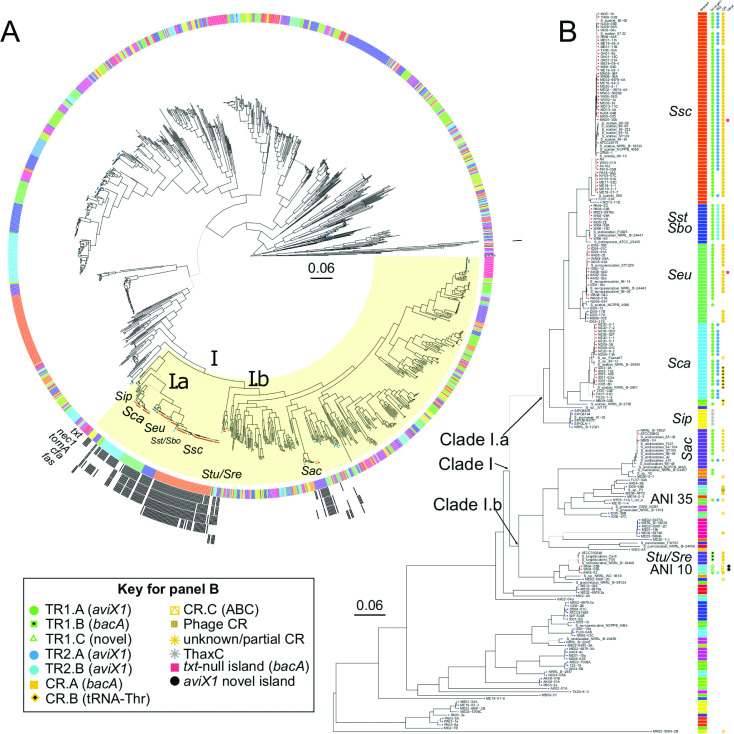
Most common scab phytopathogens belong to a single, diverse lineage of *Streptomyces *. (**a**) Core-genome phylogeny of the *Streptomyces* genus. Circle tip labels indicate the sequenced strains with red fill representing confirmed or predicted pathogens based on the presence of *txt,* blue fill representing confirmed or predicted non-pathogens based on absence of *txt*, and orange fill representing strains lacking *txt* but causing netted scab in potato infection assays. Heatmap layers surrounding the tree (from innermost out) indicate ANI group (>95 % ANI threshold), the presence of *txt* operon, *nec1*, *tomA*, *cfa* operon and *fas* operon (black indicates presence, white absence). (**b**) A MLSA tree of the phytopathogenic strains sequenced in this study and select other strains. Columns at the tip of the tree indicate ANI assignments and presence of MGEs. Lineages of key *Streptomyces* are abbreviated*: S. scabiei* (*Ssc*), *S. stelliscabiei/bottropensis* (*Sst*/*Sbo*), *S. europaeiscabiei* (*Seu*), *S. turgidiscabies/reticuliscabiei* (*Stu*/*Sre*), *S. ipomoeae* (*Sip*), *S. caniscabiei* (*Sca*) and *S. acidiscabies* (*Sac*). Potentially novel species groups are indicated with ANI group numbers. (**t**) indicates type strains of known phytopathogenic species. Trees are midpoint rooted. Branches with UFBoot >95 % and SH-aLRT >80 % are coloured black, otherwise they are coloured grey. Scale bars indicate average number of substitutions per site.

We generated and analysed genome sequences of 156 strains isolated primarily from common scab disease lesions on potato, between 2001 to 2020 and from sites in 21 US states as well as two Canadian provinces (Table S1). We additionally sequenced 10 type strains of *Streptomyces* obtained from culture collections and isolated from as early as 1961. For analyses here, we also included eight sequenced strains that are part of a novel species group [33, 40]. The sequenced strains are highly diverse and represent 45 of the species-level groups within the genus (Fig. 1; Table S2). But the distribution of sequenced strains in the genus is uneven, with most belonging to a large clade that includes *S. scabiei, S. caniscabiei, S. stelliscabiei* and *S. europaeiscabiei,* species that are known to have pathogenic members. Other sequenced strains clustered in species with known phytopathogenic members, such as *S. turgidiscabies* and *S. acidiscabies,* as well as *S. ipomoeae *. Among those in this collection, members of *S. scabiei* are the most broadly distributed across North America, with strains isolated from 13 different US states whereas those of the other three highly represented species groups (*S. caniscabiei, S. stelliscabiei* and *S. europaeiscabiei *) were identified in only four states. Strains of *S. griseiscabiei* and *S. caviscabies* were not identified among those collected in fields and were only obtained from culture collections [66, 67].

Homologues of complete sets of *txt* genes are present in 93 genome sequences of the newly sequenced strains and in 29 genome sequences retrieved from NCBI. Four additional newly sequenced genomes have homologues of the complete set of Thaxtomin C (ThaxC) biosynthetic *txt* genes. Their corresponding and potentially pathogenic strains are within the large clade labelled as ‘clade I’, which forms two sublineages, clades I.a and I.b (Fig. 1). Most of the pathogenic species, including four that clustered with *S. ipomoeae* strain 91–103, belong to clade I.a. *S. acidiscabies* and *S. turgidiscabies* form separate, distantly related lineages within sublineage clade I.b. This second sublineage also has four *txt-*bearing strains that represent two potentially novel species: strain NY05-11.A belonging to ANI (average nucleotide identity) group 35 and three other strains belong to ANI group 10 that is sister to *S. turgidiscabies *.

The distribution of *tomA* and *nec1,* as well as *cfa* (coronafacic acid) and *fas,* also implicated in virulence [31, 36, 38, 68]*,* is limited largely to members of clade I, except for one strain outside of the clade that encodes a homologue of *tomA*. Homologues of *tomA* and *nec1* are each present together in genome sequences of 102 strains. Sixteen additional strains have detectable homologs of *tomA* but not of *nec1* (Fig. 1b). However, *tomA* and/or *nec1* gene presence does not absolutely correlate with the presence of *txt* genes. Either *tomA* and/or *nec1* are found in eight strains that lack detectable *txt* homologues while 17 and 24 strains without detectable homologs of *tomA* or *nec1,* respectively, have *txt* genes. The *cfa* genes are more limited in distribution and are primarily present in *S. scabiei* and *S. turgidiscabies* (Fig. 1b). They are also found in strains that lack *txt* homologues. This cluster of genes is not known to be associated with mobile/mobilizable genetic elements. Last are the *fas* genes, which were identified only in pathogenic members of *S. turgidiscabies* and ANI group 10. In these cases, the *fas* genes are linked to *txt,* like that originally reported for *S. turgidiscabies* strain Car8 [19, 23].

### The *txt* and *tomA* genes are within multiple subtypes of MGEs

**Fig. 2.**
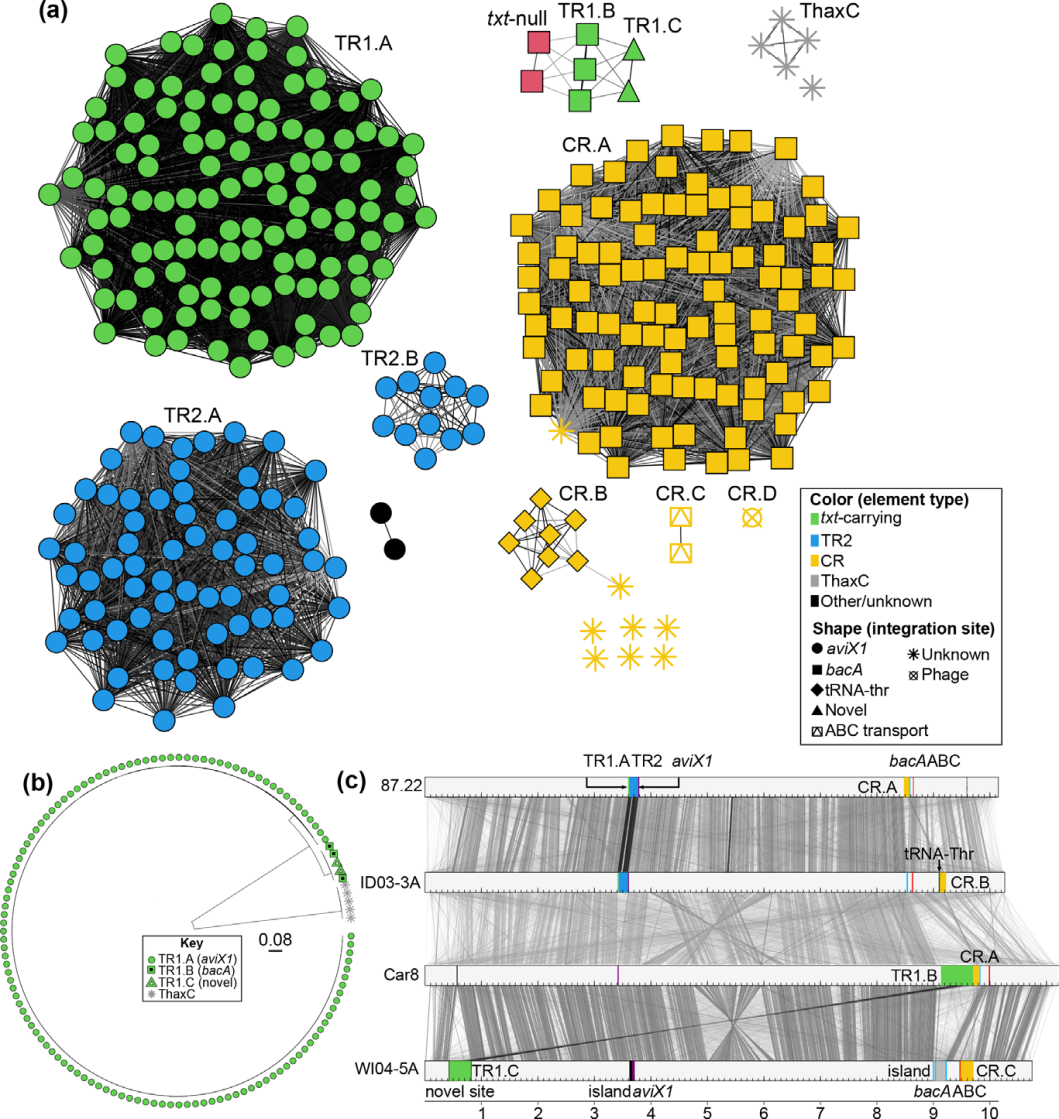
Virulence genes are associated with multiple types and subtypes of MGEs. (**a**) Weighted undirected network of MGEs. Node colour indicates the type of MGE and node shape indicates the attachment (*att*) site. Darker edges indicate greater Jaccard Index (JI) of *k*-mer signatures (minimum threshold of 0.1). (**b**) Unrooted maximum-likelihood phylogeny of *txtB* gene sequences. Tip labels indicate the type or subtype of associated MGE. Scale bar indicates average number of substitutions per site. (**c**) Genome (grey bars) alignment of strains representative of the variations in MGE types (coloured blocks) and locations (coloured vertical lines). A dashed black line indicates a partial match for an *att* site. Similarity between genomes is indicated by grey bars, darker colours indicate greater similarity. The scale at the bottom indicates increments of 1 Mb.

We next mapped virulence genes to MGEs and classified the elements into types and subtypes because they may have different evolutionary histories that need to be studied independently. We used hallmark genes, typically those implicated in virulence, to define MGE types and overall differences in sequence, composition and/or integration site to define MGE subtypes. In the genome sequences analysed here, *txt* genes (*txtH, txtR, txtA, txtB, txtC, txtD* and a cytochrome p450-encoding gene) are associated with three subtypes of TR1 IMEs or ICEs and a ThaxC type of MGE (Fig. 2a). TR1.A, as represented by that found in strain 87.22 [24], is an IME and was the most frequently identified TR1 subtype. Members formed a single, highly connected subgraph in a *k-*mer similarity network, reflecting strong similarity in sequences of conserved *txt* genes (Fig. 2a). A phylogenetic tree of *txtB* gene homologues underscores the conservation, as they clustered into one large clade with short to no branch lengths (Fig. 2b). Nonetheless, TR1.A IMEs can vary in the composition of few genes not implicated in virulence (Fig. S1).

**Fig. 3.**
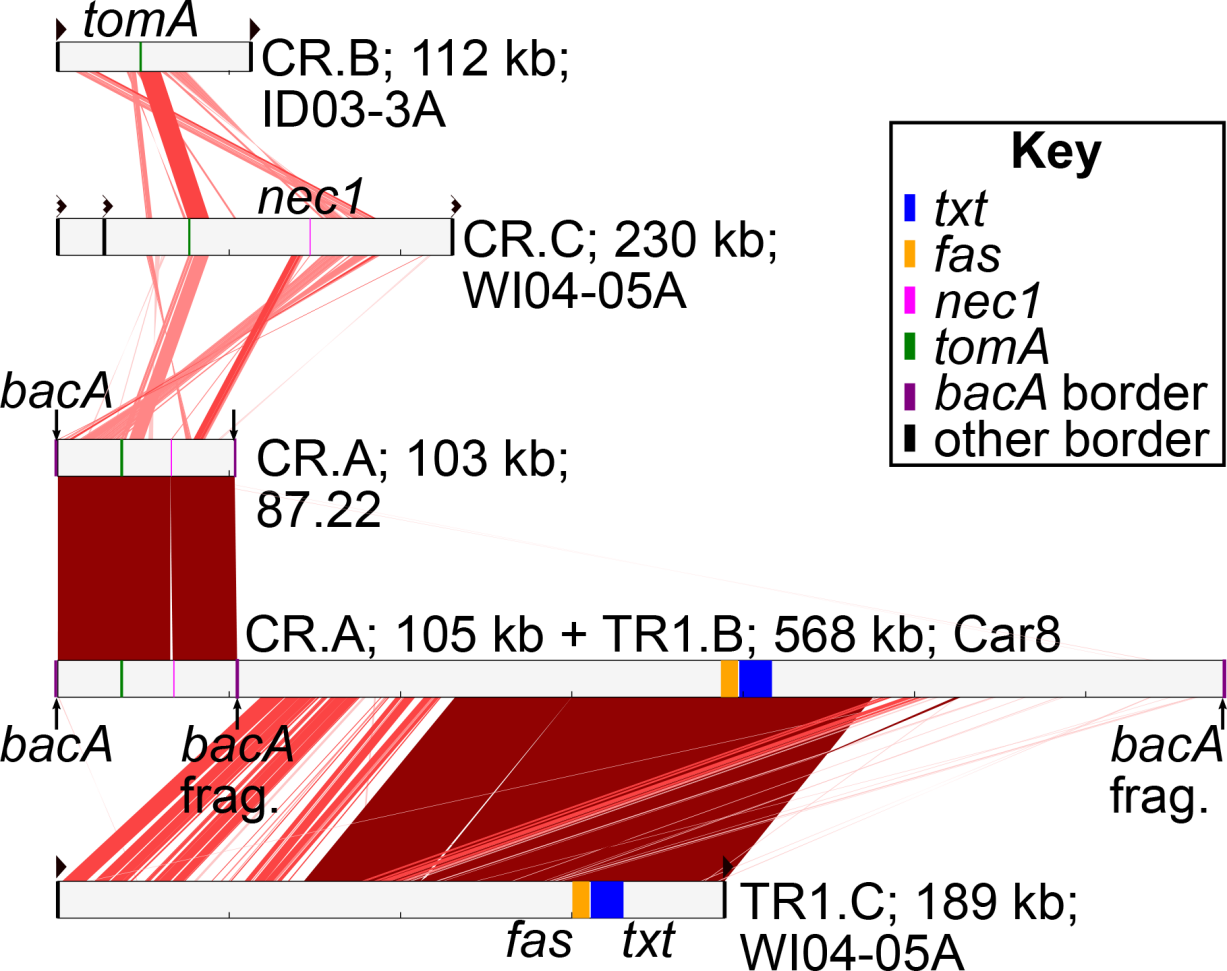
The novel TR1.C and CR.C subtypes are related to those previously characterized. Subtypes of MGEs from representative strains are depicted as light grey bars with key virulence loci or *att* sites coloured and labelled. Similarities between MGEs are indicated by red bars, darker colours indicate greater similarity. Right-facing triangles represent identifiable border sequences. Triangles with jagged edges above CR.C represent potentially degenerated border sequences.

TR1.B is an ICE and typified by the larger of the two MGEs tandemly linked within the 660 kb region of *S. turgidiscabies* strain Car8 [19, 23, 26]. TR1.B was identified in only two other sequenced strains (Figs 1b and 2a). TR1.C is an IME present only in strains WI04-05A and WI04-05B of ANI group 10 (Fig. 2a). TR1.C is similar to TR1.B, as they formed a single subgraph in the *k*-mer network (Fig. 2a). Consistent with this, their *txtB* gene homologues formed a sister clade in a phylogeny (Fig. 2b). TR1.C also has *fas* genes, but, unlike TR1.B, has different identifiable flanking border repeats, and a predicted *xerC* recombinase-encoding gene near one border (Fig. 3). TR1.C is neither tandemly integrated next to the CR region encoding *tomA* and *nec1* nor located in *bacA* or *aviX1* (Figs 2c and 3). It is unclear whether TR1.C encodes for a conjugation T4SS. Hence, TR1.C is likely a novel virulence ICE/IME and was defined as a different TR1 subtype.

All TR1.A IMEs and TR1.B ICEs are integrated in their canonical repeat sequence in *aviX1* and *bacA*, respectively (Fig. 2c). These predicted integration sites are present in nearly all analysed strains of clade I, suggesting both could be acquired by a diversity of strains. The converse appears to be the case with TR1.C, which is integrated in a region that is conserved only within *S. turgidiscabies* and ANI group 10. This region is closely related to a portion of the large element in *S. turgidiscabies* strain Car8 and while a copy of the repeat sequence could be identified in the genome sequence of strain Car8, distal to the *bacA-*associated ICE and at syntenic site as in WI04-05A, only a partial match could be found in *S. scabiei* 87.22 (Fig. 2c). We also examined whether the two strains of ANI group 10 have the genomic loci associated with the acquisition of TR1.A or TR1.B. Both genomes have canonical *aviX1* and *bacA*, but these sites are occupied by MGEs not predicted to be associated with virulence. At *aviX1,* strains WI04-05A and WI04-05B have a small island with some similarity to TR2 and at *bacA* they have tandem islands carrying genes predicted to encode diverse proteins predicted to be involved in sugar transport and catabolism (Fig. 2c).

Genes for the biosynthesis of Thaxtomin C are present in several strains, however these regions were poorly assembled likely due to the high numbers of IS elements and transposases [27]. Therefore, whether they are within MGEs and can mobilize are unclear. Nonetheless, it is notable that the *txt* genes of *S. ipomeae* are genetically diverged from those encoding for Thaxtomin A and form a separate, early branching clade in the *txtB* phylogeny (Fig. 2b).

MGEs with *tomA* and/or *nec1* genes were classified as one of four subtypes of CR (Figs 2 and 3). More than 95 % of the CR members have a conserved region that includes *tomA* and roughly 16 surrounding genes (Fig. S2; [26]). The *tomA* homologues are conserved within a CR subtype but are diverged in sequence across the subtypes (Fig. S3). No T4SS-encoding genes were detected in this region nor outside of it; therefore, we considered CR elements as IMEs. It was previously suggested that these elements also lack an integrase-encoding gene [26], but we identified a subset with a gene bordering one *att* site and predicted to encode a functional integrase. CR.A is represented by the element found in *S. scabiei* [24] and the homologous region of the modular ICE of *S. turgidiscabies* [19, 23]. This subtype is broadly distributed across members of clade I (Fig. 1b). CR.A is located in *bacA* and is the most common subtype. CR.B is common in the *S. caniscabiei* species group and integrated near a tRNA-Thr encoding gene (Figs 1b and 2c). This subtype lacks detectable homologues of *nec1* (Fig. S3). CR.C carries both *tomA* and *nec1* and is a novel subtype in strains WI04-05A and WI04-05B of ANI group 10 (Fig. 2a). The *nec1* homologue of this subtype is diverged from that present in CR.A (Fig. S3). CR.C is integrated in an ABC transporter-encoding gene but flanked by IS elements and with one degenerated border sequence, potentially precluding it from further recombination (Figs 2c and 3). Last, and surprisingly, CR.D is located between two predicted partial phage loci in strain *Streptomyces* sp. P3. CR.D has no identified border sequences and could have been mobilized by phages (Fig. 2a).

### *Streptomyces* have multiple subtypes of non-virulence MGEs implicated in mobilizing virulence MGEs

We next used compositional differences to classify the TR2 MGEs that are linked to the TR1 subtypes [25, 69]. All TR2 members were predicted to be ICEs based on having gene homologues encoding for integration/excision and conjugation; but because of compositional variation, formed two subtypes (Figs 2a and S4). Also, as described above, we identified a small island within the *aviX1* site of the two ANI group 10 strains, which have TR1.C distal to *aviX1* (Figs 2c and S4). These islands have low similarity to TR2, lack genes for integration or conjugation, but have conserved border sequences.

In this dataset, the TR2 subtypes are associated with different species of *Streptomyces *, with TR2.A present in *S. scabiei, S. caniscabiei*, *S. acidiscabies* and ANI group 35 (Fig. 1b). Also, we noted that TR2.A ICEs are linked to only one single nucleotide polymorphism (SNP) variant of TR1.A (Fig. S5) and it is possible that the TR2.A ICEs may have mobilized this specific variant across species groups. Consistent with previous observations [24], in *S. scabiei* and *S. caniscabiei *, TR2 is not always associated with TR1, suggesting the ICE can be lost. Conversely, it was previously suggested that loss of a TR2 subtype was more ancestral in *S. acidiscabies* species [24], but in this dataset, TR2.A is present in the genome sequence of one *S. acidiscabies* strain. This either reflects recent loss of TR2.A in some strains of *S. acidiscabies* or gain in strain a10. The TR2.B subtype is limited in this dataset to only *S. stelliscabiei* and one strain in ANI group 10 but is tandemly inserted next to one of four different TR1.A variants (Fig. S5). No TR2 subtype was found among the sequenced *S. europaeiscabiei* strains and loss from this species may have been more ancestral (Fig. 1b). TR2 is not present in any strain that lacks TR1.A (Fig. 1 **,** Table S1).

*S. scabiei* strain MN05-35A and *S. europaeiscabiei* strain AK08-04D have homologous and unique MGEs, here classified as *txt*-null ICEs, that are closely related to TR1.B and integrated in *bacA* adjacent to a CR.A IME (Figs 1b, 2a and S6). However, the *txt-*null ICEs lack virulence genes implicated in common scab disease, but the integrase- and relaxase-encoding genes are homologous to those of TR1.B (Figs S6 and S2). There is potential the ancestor of the *txt-*null ICE acquired a large region with *txt* genes and is a progenitor of the modular MGE of *S. turgidiscabies* (Fig. S6). Alternatively, the *txt*-null ICE is derived from the modular MGE but has since lost the virulence region. Regardless, given the presence of genes necessary for recombination, it is possible these ICEs mobilize *bacA*-integrated IMEs.

### MGE subtypes have different evolutionary histories

**Fig. 4.**
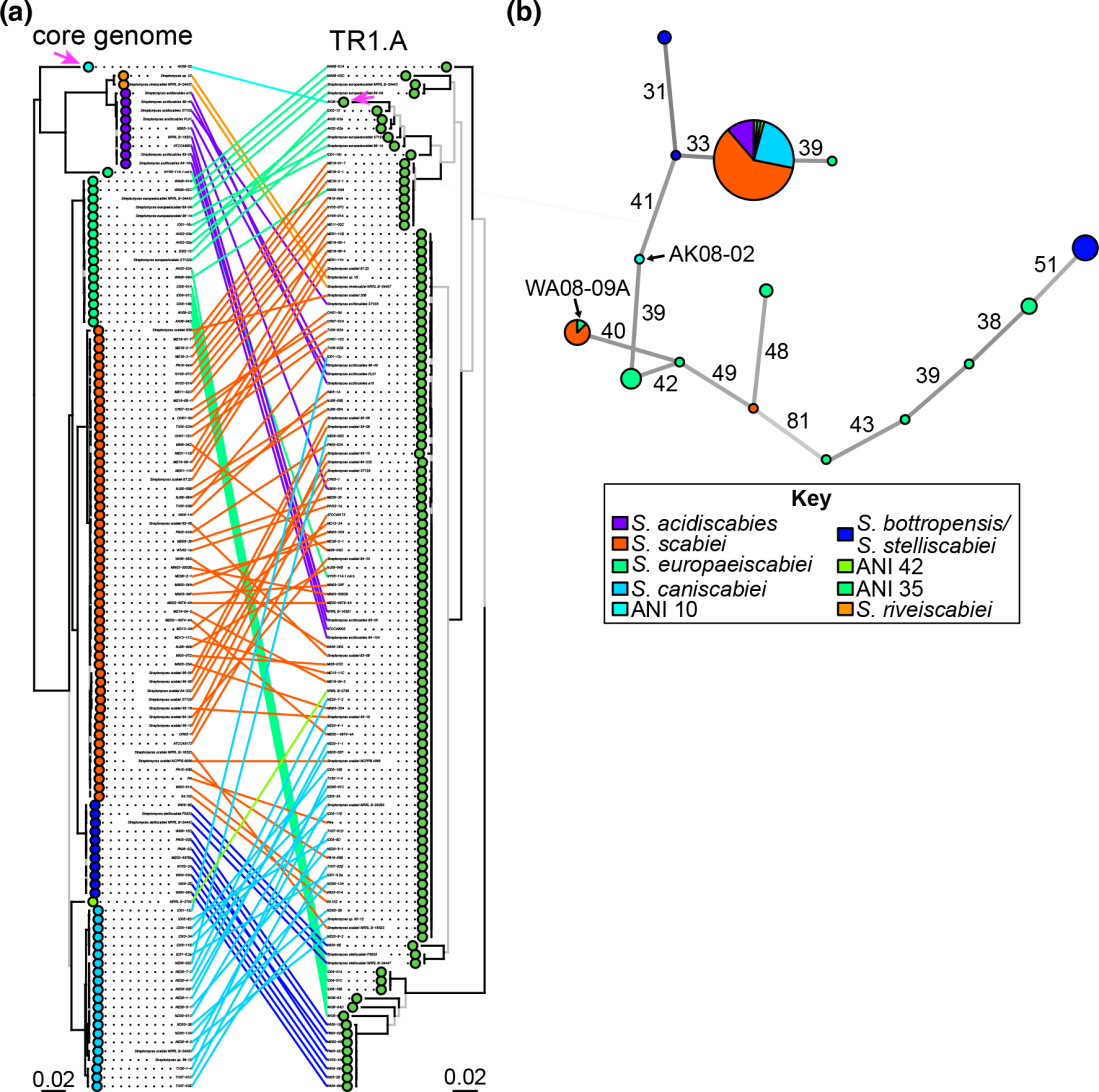
TR1.A has been horizontally transferred among *Streptomyces* species groups. (**a**) Co-phylogeny plot comparing the evolutionary history of strains (left; core genome tree) and TR1.A SNP variants (right). In the core genome tree, tips are coloured by ANI group. Lines connect strains to their corresponding MGE and magenta-coloured arrows highlight AK08-02 and its TR1.A. Clades of identical sequences in the TR1.A tree are arbitrarily ordered. The trees are midpoint rooted. Branches with UFBoot >95 % and SH-aLRT >80 % are coloured black, otherwise coloured grey. Scale bars indicate average number of substitutions per site. (**b**) Minimum spanning network of TR1.A IMEs. Nodes representing SNP variants of TR1.A are scaled according to the number of variants (smallest is one member; largest is 82 members). Colours in nodes represent the species or ANI group designation of the strains associated with the SNP variant. Edges linking nodes are labelled with the number of pairwise SNP differences between types. Darker coloured edges indicate fewer SNP differences.

We next searched for evidence of HGT of individual subtypes. Previous methods used to support HGT relied on identifying strains diverged in chromosomal sequence but with *txt* genes highly similar in sequence [25, 39]. Closely related strains that have highly diverged *txt* genes would also be consistent with HGT. In this dataset, examples consistent with the former case are the high degree of similarity between *txt* genes of *S. acidiscabies* and ANI group 35 compared to the *txt* genes in the more distantly related strains of clade I.a. However, all *txt* homologues identified in our dataset are extremely similar in sequence (Fig. 2b). Low diversity could also reflect strong purifying selection as opposed to HGT, which limits the ability to identify examples that support either of the scenarios. Therefore, we queried the dataset for incongruencies in core genome and *txtB* gene phylogenies that were also supported by differences in TR1 subtypes. We identified only one instance (Fig. 4a). Strain AK08-02 has a canonical TR1.A IME located in *aviX1*, but the strain is a member of ANI group 10 (Fig. 1), in which the other two members carry a TR1.C ICE (Fig. 3). Notably, strain AK08-02 also has TR2.B while other members of ANI group 10 do not, suggesting that both IME and ICE were acquired horizontally relatively recently. In contrast to gains, recent loss of TR1 subtypes is supported by this dataset to have occurred frequently in *Streptomyces *. No *txt* homologues were detected in 77 of the newly sequenced strains, including several strains within typical pathogen species in clades I.a and I.b. Most notably, two *S. caniscabiei* strains, four *S. scabiei* strains and six *S. europaeiscabiei* strains included in this analysis lack *txt* genes likely due to recent loss (Fig. 1b).

In a second approach, we focused exclusively on TR1.A, and evaluated the distribution of SNP variants of this IME for a higher resolution investigation into patterns indicative of HGT, as has been used in other studies on subsets of highly similar MGEs [8, 9, 70] (Fig. 4a, b). The most common SNP variant is in 82 strains and distributed across multiple species groups of clade I.a. It is also present in *S. acidiscabies* and ANI group 10 (strain AK08-02) of clade I.b. This is consistent with previous models suggesting HGT [24, 26, 39]. We also looked for evidence of closely related strains with diverse TR1.A variants. Strains of *S. europaeiscabiei* carry multiple variants of TR1.A (Fig. 4a, b). Strains of *S. scabiei* and *S. stelliscabiei* also carry at least two variants of TR1.A. Together these suggest that these species groups have acquired the TR1.A multiple times or that TR1.A diversified within one of these species groups. Other lineages are more restricted. Strains of *S. caniscabiei* and *S. acidiscabies* only carry one variant of TR1.A. It is possible that these lineages acquired the TR1 in a single event and that it has been largely vertically inherited since then.

In a third approach, we focused on the rare SNP variants to infer HGT because TR1.A is small with few informative sites, again making it difficult to differentiate from strong purifying selection. This approach further supported HGT of the TR1.A IME by AK08-02 (Fig. 4b). Another HGT event was predicted for strain WA08-09A of the *S. europaeiscabiei* species, which has 10 different SNP variants of TR1.A IMEs. The TR1.A SNP variant of strain WA08-09A grouped with a subset of those in *S. scabiei* strains (Fig. 4b). However, it is important to note that none of these predicted HGT events is associated with the emergence of new phytopathogenic species of *Streptomyces *. Both patterns reveal incongruencies among taxa that have different subtypes of TR1 or SNP variants of TR1.A and indicate that HGT led to the swapping of MGEs but did not alter the phytopathogenicity trait in recipient cells.

The *tomA* gene has stronger evidence for more frequent mobilization. The conservation of *tomA* and *nec1* homologues within CR subtypes is consistent with ancient events transferring these virulence genes into different elements, followed by their divergence. We found no evidence that homologues have been swapped between subtypes since divergence. But there is at least one instance, with strain AK08-02, in which a subtype can be inferred to have been acquired by HGT. We also examined SNP variants of the CR.A subtype to find evidence for HGT events of MGEs that occurred relatively more recently in evolutionary time (Fig. S7A). CR.A formed three main lineages, each with multiple SNP variants. Notably, all three lineages (CR.A1, CR.A1, CR.A3) are present within *S. europaeiscabiei,* suggesting its members independently and relatively recently acquired CR.A or that diversification of CR.A occurred with *S. europaeiscabiei* (Fig. S7). CR.A1 and CR.A2 are also present in both *S. scabiei and S. acidiscabies*, suggesting that these IMEs have been acquired multiple times in their evolutionary history. Alternatively, *S. stelliscabiei* strains have, exclusively, closely related CR.A1 MGEs (Fig. S7B). This suggests that this species group may have acquired CR.A once, and it was subsequently vertically inherited. Another well-supported example of HGT of CR.A is found in strains AK08-03 and AK02-02A. These two *S. europaeiscabiei* strains are extremely closely related, with chromosomes (excluding ICEs/IMEs) separated by only 280 SNP differences, but they carry different CR.A lineages (Fig. S7B). The CR.A IMEs tandem to TR1.B ICEs in *S. turgidiscabies* strains are closely related to CR.A variants found in *S. scabiei* strains, suggesting these may have been acquired horizontally (Fig. S7). Last, analysis of SNP variants cross-validated the predicted HGT that involved strain AK08-02. Altogether, findings suggested that *tomA* and linked genes have been mobilized frequently in the history of *Streptomyces *. However, it is generally the case that once acquired, CR is maintained by vertical inheritance.

### *Streptomyces* strains vary in virulence within species and ICE/IME subtypes

**Fig. 5.**
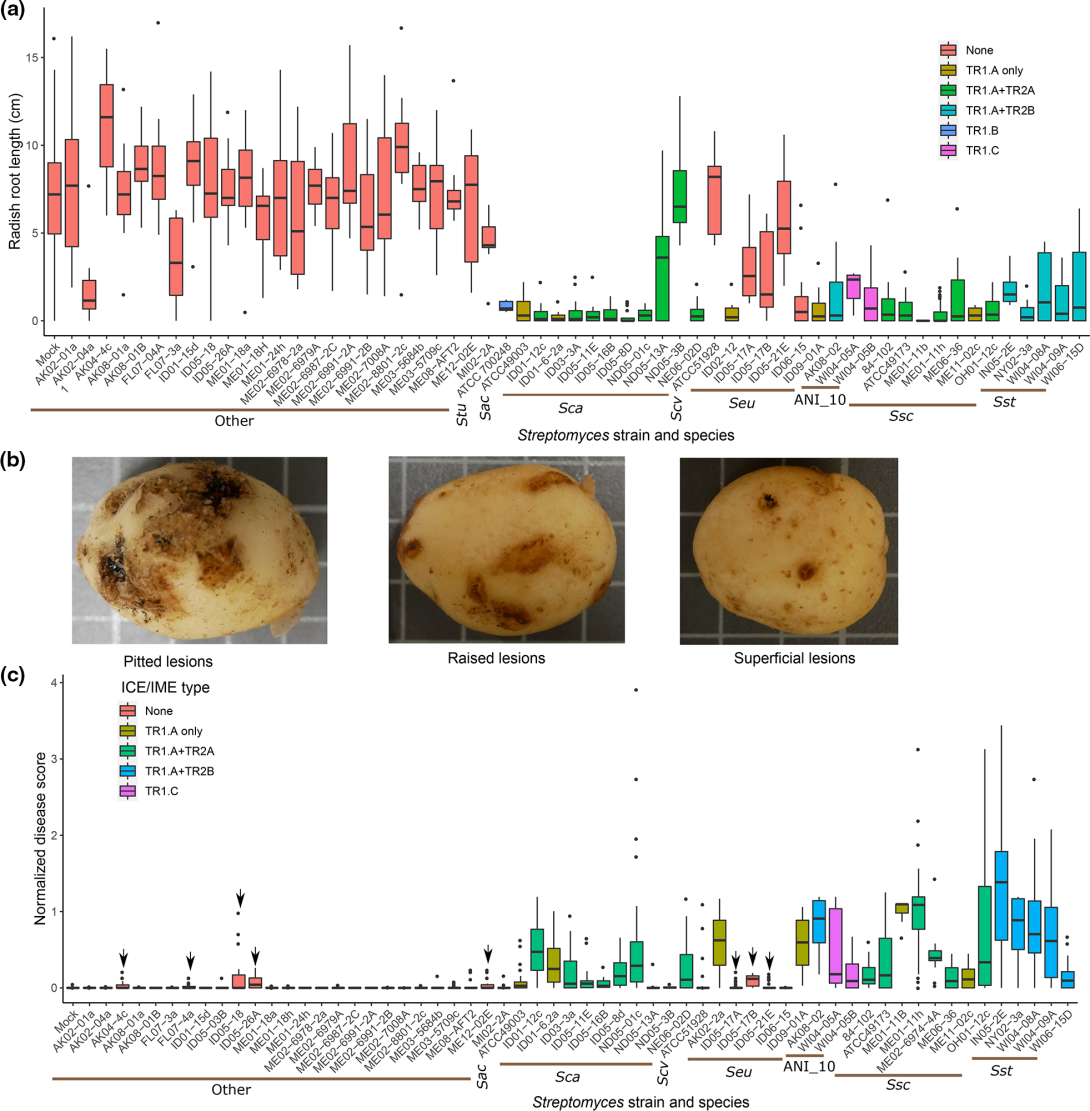
Neither species designation nor ICE/IME subtype explain the variation in virulence of *Streptomyces* strains. (**a**) Stunting of radish seedlings, as measured by root length, following inoculation with mycelial mass of indicated *Streptomyces* strains for 9 days. *n*=12 individual plants for each strain. (**b**) Example of distinct lesion types used to define common scab disease severity on potato tubers. (**c**) Normalized disease severity on potato *cv*. Chipewa tubers following inoculation with indicated *Streptomyces* strains. All tubers were scored from three replicate pots 12 weeks after planting and inoculation. Arrows indicate strains that caused superficial lesions only (indicative of netted, not common, scab) in more than one replicate pot. Species abbreviations: *Sac, S. acidiscabies; Sca, S. caniscabiei; Scv, S. caviscabies; Seu, S. europaeiscabiei; Ssc, S. scabiei; Sst, S. stelliscabiei*.

With a clearer understanding of variations and relationships among virulence gene-associated MGEs, we tested whether specific subtypes or combinations of MGEs influenced virulence. We selected 60 *Streptomyces* strains representing seven different phytopathogenic species and included 26 strains lacking *txt* genes for plant infections. When inoculated onto radish seedlings, all strains with *txt* genes caused significant plant stunting, as measured by attenuated root length (Fig. 5a), hypocotyl length (Fig. S8A) and cotyledon cross section (Fig. S8B). For all tested species with *txt-*containing members, there was a wide range in the degree of radish stunting with species group, strain, CR element subtype and *txt* element subtype being significant factors for hypocotyl length based on Krukal–Wallis tests (Table S3). But there was no clear correlation between degree of stunting and *txt* element subtype (Fig. 5a) or CR subtype (Fig. S9A) among the TR1-containing strains.

On potato, only strains with *txt* genes were able to cause raised and pitted lesions indicative of common scab, though several strains lacking *txt* genes were able to cause superficial lesions associated with netted scab (Fig. 5b, c). Only three of the tested strains that cause netted scab, the three *S. europaeiscabiei* strains, encode a CR (Fig. S9B). For each of the seven tested species with phytopathogenic members with multiple strains tested*,* there is a wide range of disease severity caused by individual strains within the species (Fig. 5c) indicating that the species group is alone not predictive of strain virulence. However, based on Kruskal–Wallis non-parametric tests, there were significant differences in disease scores across species, strain, CR subtype and TR subtype (Table S3).

Strains within the same species groups with different subtypes of *txt* or CR elements may provide preliminary indication if specific virulence gene subtypes are associated with increased virulence. As expected, presence/absence variation of TR2.A in *S. caniscabiei* and *S. scabiei* was not predictive of strain virulence (Fig. 5c). A similar observation was made for CR.B and CR.A of *S. caniscabiei* and *S. scabiei *, respectively (Fig. S9B). In fact, strains in these species groups and lacking CR were among the most virulent of the tested strains. For ANI group 10, one strain, AK08-02, has TR1.A and a CR.A IME and was significantly more pathogenic than the other two strains with TR1.C and CR.C. (Fig. 5c). However, given the low number of strains tested and limited available strains within this species-level group, it is difficult to link phenotypic differences to differences in MGE composition. Taken together, the potato disease results confirm the necessity of the *txt* ICE or IME for common scab of potato but suggest that neither the TR1 subtype nor presence/absence of the CR are predictive of the degree of strain virulence.

### Recent dispersal of common scab pathogens is rare

**Fig. 6.**
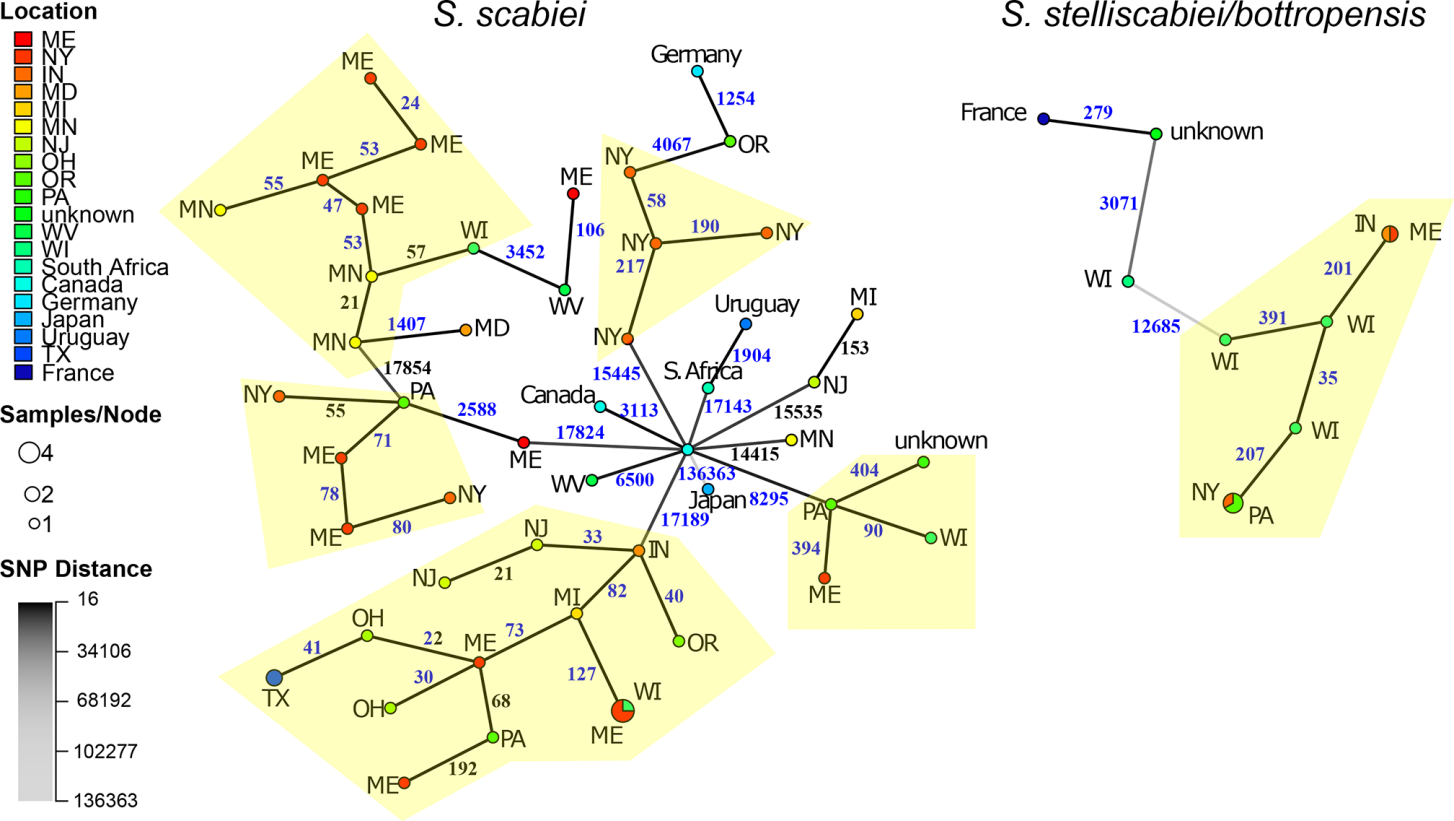
Pathogenic *Streptomyces* was dispersed historically and recently. Minimum spanning network of the most deeply sampled phytopathogenic species-level groups, (**a**) *S. scabiei* and (**b**) *S. stelliscabiei */*bottropensis* strains. Nodes represent genotypes and are scaled to represent the number of members (smallest is one member; largest is four members). Clusters of closely related genotypes are highlighted in yellow. Colours in nodes represent locations of fields from which strains were isolated. Edges linking nodes are labelled with the number of pairwise SNP differences between genotypes. Darker coloured edges indicate fewer SNP differences.

In the last of our analyses, we examined the deeply sampled species-level groups of pathogenic *Streptomyces* for epidemiological patterns to understand the contribution of pathogen spread in the evolution of phytopathogenic *Streptomyces* (Fig. 6, Tables S4−S10). Two of the species-level groups, *S. scabiei* and *S. stelliscabiei *, exhibited patterns consistent with recent dispersal of pathogenic strains. Within both groups*,* genotypes are diverse and were related by as few as 21 SNP differences to more than 130 000 SNP differences. Minimum spanning networks revealed two important observations. First, locations linked by clonal strains were rare, as there were only four genotypes represented by more than one strain. The two best supported links were *S. stelliscabiei* strains IN05-2E and ME02-6978b, isolated from Indiana and Maine, respectively, and strains NY02-3A and PA06-02B isolated from New York and Pennsylvania, respectively (Fig. 6b, multicoloured circles). There were also two links in *S. scabiei,* but neither are strong examples. One was not indicative of a transmission event because strains of the same genotype were isolated from the same field in Texas. The second case includes three strains isolated from the same field in Maine and reference strain *S. scabiei* 87.22, isolated from Wisconsin (Fig. 6a). Records showed that the field site in Maine from which these strains were isolated is a research farm that contained trials with seed pieces artificially inoculated with unspecified *Streptomyces scabiei* strains in 1989 and 1990 [71]. Therefore, there is potential that this epidemiological link is due to experimental inoculation.

The second observation revealed by the minimum spanning network is that despite there being few supported cases of clonal spread of pathogens, many of the genotypes are closely related. Notably, there is a group of strains of *S. scabiei* isolated from six different states that differ by fewer than 200 SNP differences. We also identified several smaller clusters of closely related strains of *S. scabiei* and *S. stelliscabiei* with members present across different states (Fig. 6, yellow highlighting). Conversely, *S. scabiei* strains isolated from countries outside of North America were separated by thousands of SNP differences.

## Discussion

Virulence is a complex trait that in *Streptomyces* likely reflects interactions between *txt, tomA* and other genes [21, 22, 32]. To generate a detailed understanding on the evolution of phytopathogenicity, we systematically deconstructed the *Streptomyces-*potato pathosystem. We integrated findings from the analyses of virulence genes and their associated MGEs as well as analyses of bacterial diversity and pathogen spread. We showed that *txt* genes are extremely conserved in sequence while *tomA* homologues are more diverse in sequence (Figs 2 and 3). But homologues of the two are distributed across diverse types and subtypes of MGEs, each with unique histories that have shaped the evolution of *Streptomyces* differently. Findings also showed that there is substantial taxonomic diversity of *Streptomyces* associated with potato fields and new species groups are still being discovered. However, strains inferred to be pathogenic are limited to clade I.a and few species-level groups in clade I.b (Fig. 1). Surprisingly, neither species group nor virulence gens ICE/IME subtype was predictive of an individual strain’s phytopathogenicity (Fig. 5).

The association of virulence genes to MGEs has been well documented and led to models that predicted HGT as a major force in driving the emergence of new phytopathogenic *Streptomyces* lineages [25, 39]. However, these models were low on resolution because analyses were limited to few genes and pathogenic strains. The since improved capacity to sequence and analyse populations of bacteria has helped to dramatically shift our perspective and show that MGEs provide multiple mechanisms for disseminating traits. In clinical settings, clonal expansion of antibiotic resistant lineages is commonly observed and can lead to global spread of vertically inherited MGEs [72, 73]. Antibiotic resistant genes also spread to new MGEs, increasing potential for horizontal transmission within and across taxonomic boundaries [74]. Importantly, and particularly with complex traits, recombination among MGEs can diversify traits [41]. These mechanisms collectively contribute to both diversification and robustness of traits. Prior to the study here, the contributions of these and other possible mechanisms on the emergence of phytopathogenicity in *Streptomyces *, one of the major pathogens of potato, was unknown.

Ancient HGT events had a significant role in the evolution *Streptomyces* phytopathogenicity. The earliest HGT events involving *txt* are inferred to be independent acquisitions of an ancestorial TR1.A/B/C and ThaxC, the latter of which in this dataset, is found only in *S. ipomoeae *. It is also possible that very early divergence of ThaxC from TR1 and subsequent vertical inheritance by *S. ipomoeae* explains the distinction between these two MGEs, as previously proposed [39]. The next was HGT of *txt* and divergence into TR1.A and the ancestor of TR1.B/C. A relatively more recent HGT of *txt* led to the separation of TR1.B ICE and TR1.C IME. A potentially more recent HGT event is supported by the observation that TR1.A is present in both clade I.a strains and the more distantly related clade I.b strains (strains within *S. acidiscabies and* ANI group 35), indicative of HGT of this IME, as previously suggested [39].

In disagreement with current models, within our dataset, there are few supported examples of very recent HGT of *txt*, all of which involve TR1.A (Fig. 4). The best supported examples of HGT of *txt* elements involve *S. europaeiscabiei* strain WA08-09A having a SNP variant of TR1.A otherwise only found in *S. scabiei *, and ANI species group 10 strain AK08-02 having TR1.A while the other two ANI species group 10 strains have TR1.C. But neither of these events can be implicated with the emergence of new lineages. Rather, they are inferred to involve swapping of SNP variants of TR1.A among pathogenic groups. Moreover, while previously models suggested that the polyphyletic distribution of TR1 is evidence of rampant ancestral HGT of TR1, we propose that frequent losses of TR1.A IMEs has led to the polyphyletic distribution of *txt* genes (Fig. 1). Because TR1.A is an IME, its integration is dependent upon a TR2 ICE and maintenance could be less stable.

HGT of *tomA* appears to have occurred recurrently throughout the history of the genus. Ancestral HGT led to *tomA* being within at least four different subtypes of CR elements and subsequent virulence gene divergence. More recent exchange has distributed CR.A across *Streptomyces *, as this subtype is present not only in clade 1.a but also more broadly in clade 1.b. There is potential that a subset of CR.A are functional ICEs. Moreover, within clade I.a, there is evidence for evolutionary recent HGT, including acquisition of new subtypes and SNP variants. Despite that *tomA* and the CR elements persist in the population and are frequently with *txt* genes in genomes, their roles in virulence remain unresolved. CR elements are not always present in lineages that cause common scab disease and some strains lacking such elements are some of the more virulent among those tested. There is potential for CR to affect host specificity, as cultivar by strain interactions significantly affect disease outcomes [65]. Here, a single highly susceptible potato cultivar was used as a host. It is also likely that virulence is impacted by genes outside of the analysed MGEs. Last, it is possible that different combinations of *txt* and *tomA* alleles could influence virulence.

The transfer of *txt* and *tomA* to different subtypes of MGEs is advantageous because of variations in modes of transmission, integration and bacterial host range. For example, *txt* genes are broadly distributed because they are associated with subtypes of elements that are collectively compatible with diverse lineages of *Streptomyces *. The *tomA* gene is associated with relatively large (>100 kb) MGEs, which integrate into different sites of chromosomes (Fig. 2c). It was previously suggested that genomes and bacteriophages have co-evolved to avoid disruption while allowing recombination [75]. Similarly, it is suggested that mega-sized symbiosis ICEs vary in integrases that provide flexibility in matching with certain chromosomes [10]. Hence, it is conceivable that CR subtypes may be co-adapted to specific *Streptomyces* chromosomes. It is also notable that *tomA* can be uncoupled from the large CR element and be potentially mobilized by bacteriophages.

Regarding the spread of the pathogen, analyses uncovered only a few examples of epidemiological links among phytopathogenic *Streptomyces* genotypes, some of which may have been caused by experimental inoculations of fields (Fig. 6). Conversely, in North America, many subsets of *S. scabiei* genotypes are separated by few SNP differences, yet are broadly distributed across fields. Importantly, North American strains are separated by many SNP differences relative to strains isolated in different countries. Hence, the similarity among North American fields suggested that spread of *S. scabiei* did occur frequently and recently in modern times. But, the paucity of evidence for clonal spread suggested that pathogen dispersal has since been largely curtailed. In the early 1900s, the United States and Canada adopted a certification programme to mitigate the spread of disease on seed potatoes [76]. The establishment of inspection agencies and continual advancement in efforts to distribute exclusively clean seed tubers may have reduced the spread of common scab because the obvious appearance of common scab symptoms allows for easy removal of infected seed material prior to shipping. We propose that prior to these efforts, pathogenic *Streptomyces* was frequently spread and led to establishment of lineages in fields. A deep samping of fields is necessary to better inform on the diveristy within sites and model the roles of HGT on diversifying potentially isolated populations.

In summary, HGT of *txt* and *tomA* virulence genes was an important but mostly ancient innovation in *Streptomyces *. These virulence genes have been shuffled across different subtypes of elements, which has helped spread them broadly across the genus. However, more recently, vertical inheritance and losses are inferred to have major roles in shaping phytopathogenicity. Similarly, in the past, pathogenic strains were dispersed broadly across North America. But more recently, control measures appear to have limited spread but because of earlier practices, pathogenic lineages are endemic to fields.

## Supplementary Data

Supplementary material 1

Supplementary material 2
