# Supplementary material for: Diverse mobile genetic elements shaped the evolution of Streptomyces virulence
Source: Microb Genom. 2023 Nov 6;9(11):001127. doi: 10.1099/mgen.0.001127 (PMC10711302; doi:10.1099/mgen.0.001127)
Supplement: Supplementary material 1 [file mgen-9-1127-s001.pdf]

Supplemental files for the manuscript:

"Diverse mobile genetic elements shaped the evolution of *Streptomyces* virulence"

Author names

Alexandra J. Weisberg[1], Emma Pearce[1], Charles G. Kramer[2], Jeff H. Chang[1]*, and Christopher R. Clarke[2]*

Affiliation(s)

[1]Department of Botany and Plant Pathology, Oregon State University, Corvallis, OR 97331 USA

[2]USDA Agricultural Research Service, Genetic Improvement for Fruits and Vegetables Lab, Beltsville, MD, United States

Corresponding author and email address

Correspondence to: Christopher.clarke@usda.gov or changj@oregonstate.edu

Supplemental table legends.

Table S1. Genomes sequenced in this work and strain metadata.

Table S2. Average nucleotide identity of all genome sequences analyzed.

Table S3. Kruskal-Wallis non-parametric tests for pathogenicity on radish and potato datasets.

Table S4. Pairwise single nucleotide polymorphisms for *S. acidiscabies* strains.

Table S5. Pairwise single nucleotide polymorphisms for *S. stelliscabiei/bottropensis* strains.

Table S6. Pairwise single nucleotide polymorphisms for *S. caniscabiei* strains.

Table S7. Pairwise single nucleotide polymorphisms for *S. europaeiscabiei* strains.

Table S8. Pairwise single nucleotide polymorphisms for *S. scabiei* strains.

Table S9. Pairwise single nucleotide polymorphisms for *Streptomyces* ANI group 10 strains.

Table S10. Pairwise single nucleotide polymorphisms for *S. turgidiscabies* strains.

Supplemental figures and legends

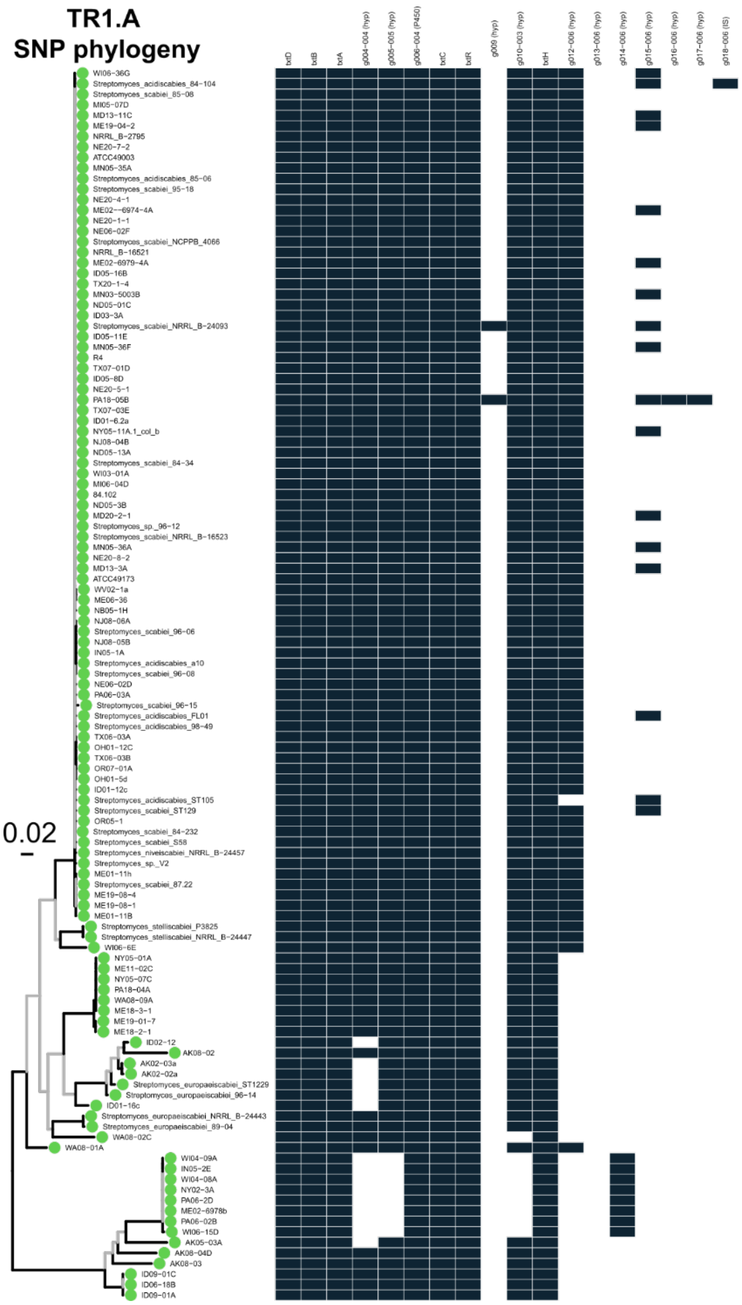

Figure S1. TR1.A IMEs are conserved in sequence and gene content. The phylogenetic tree is based on SNP differences among TR1.A IMEs. The heatmap at the tip of the tree shows gene presence/absence patterns of gene homologs. Presence of a homolog is indicated by a black box while absence is indicated by a white box. The tree is midpoint rooted. Branches with UFBoot > 95% and SH-aLRT > 80% are colored black, otherwise colored gray. Scale bars indicate the average number of substitutions per site.

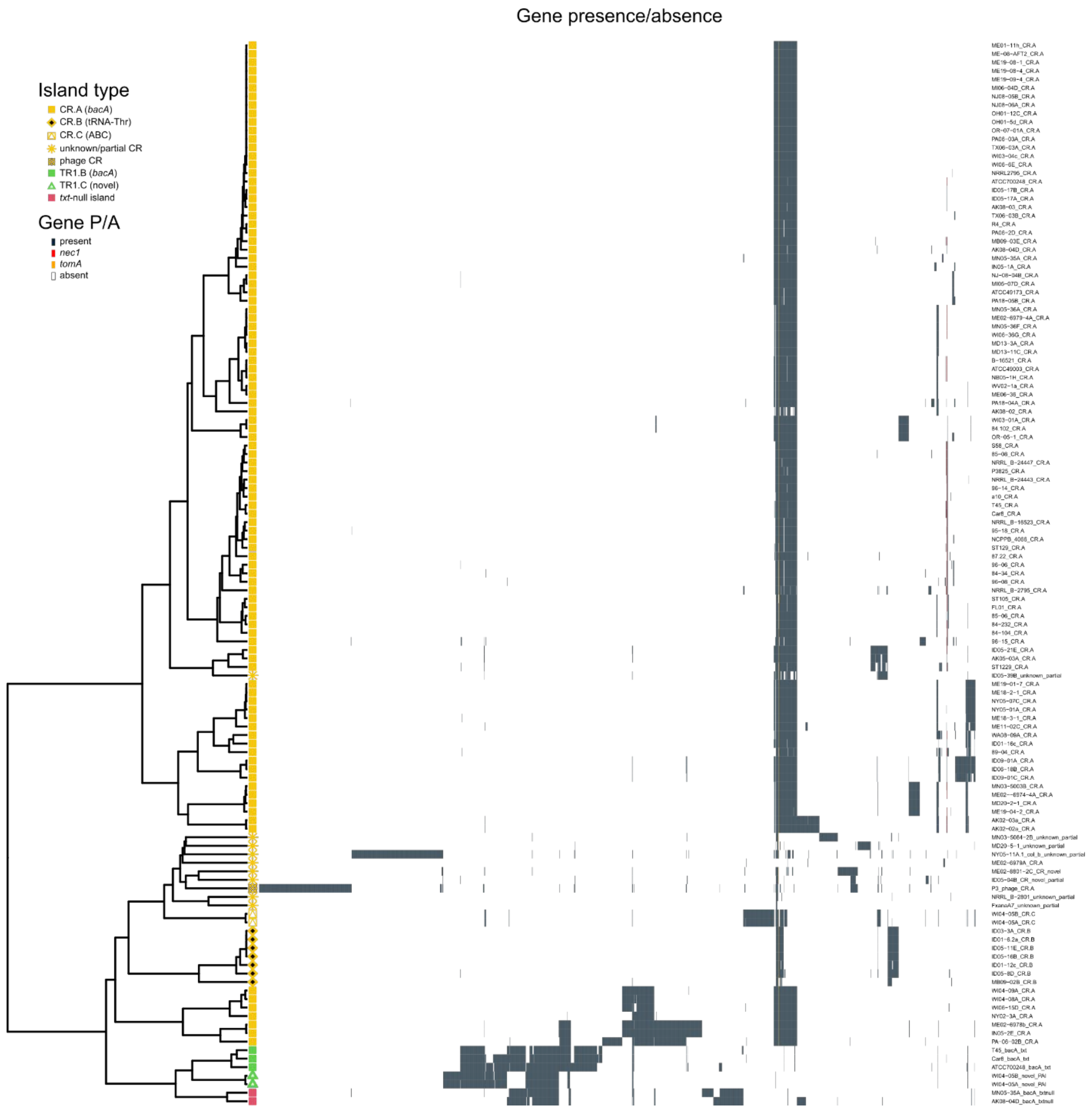

Figure S2. The CR type includes multiple subtypes. A dendrogram based on row clustering is shown on the left. The next column shows the type of CR MGE present in the strain. Columns of the heatmap show presence (black box) and absence (white box) of homologs. The presence of *tomA* and *nec1* are highlighted in color. Hierarchical clustering was based on ward.D2 clustering of binary distances. A dendrogram based on column clustering is not shown.

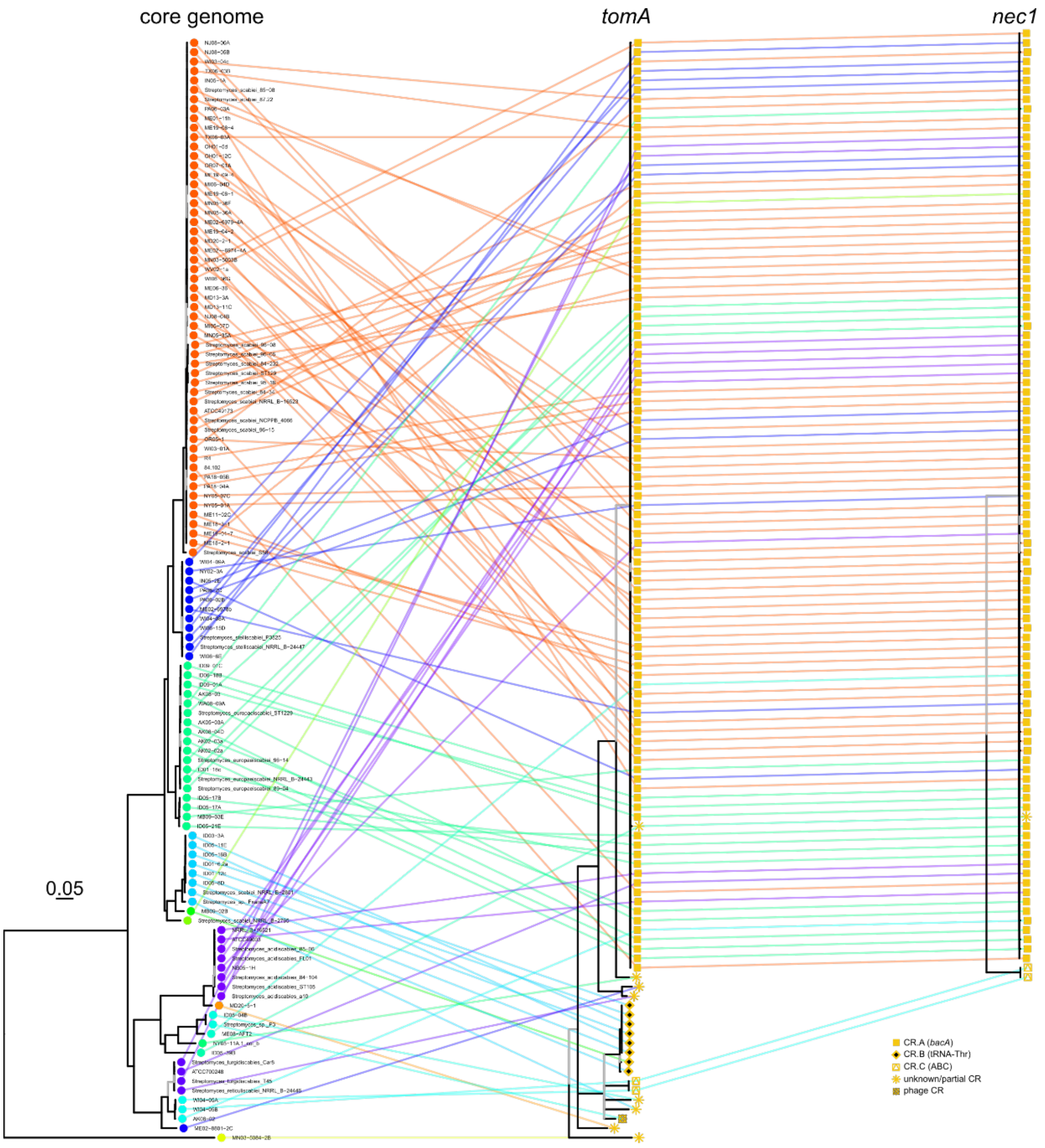

Figure S3. The *tomA* and *nec1* alleles are associated with different CR subtypes. Multi co-phylogeny plot comparing the evolutionary history of strains (core genome tree), *tomA*, and *nec1*. Lines connect strains to their corresponding genes. For the core genome tree, tips are colored according to ANI or species group (core genome tree). The large clades of identical sequences in *tomA* and *nec1* trees are arbitrarily ordered. For the two gene tree, symbols at the tips indicate the subtype of MGE. Trees are midpoint rooted. Branches with UFBoot > 95% and SH-aLRT > 80% are colored black, otherwise colored gray. The scale bar applies to all three trees and indicates average number of substitutions per site.

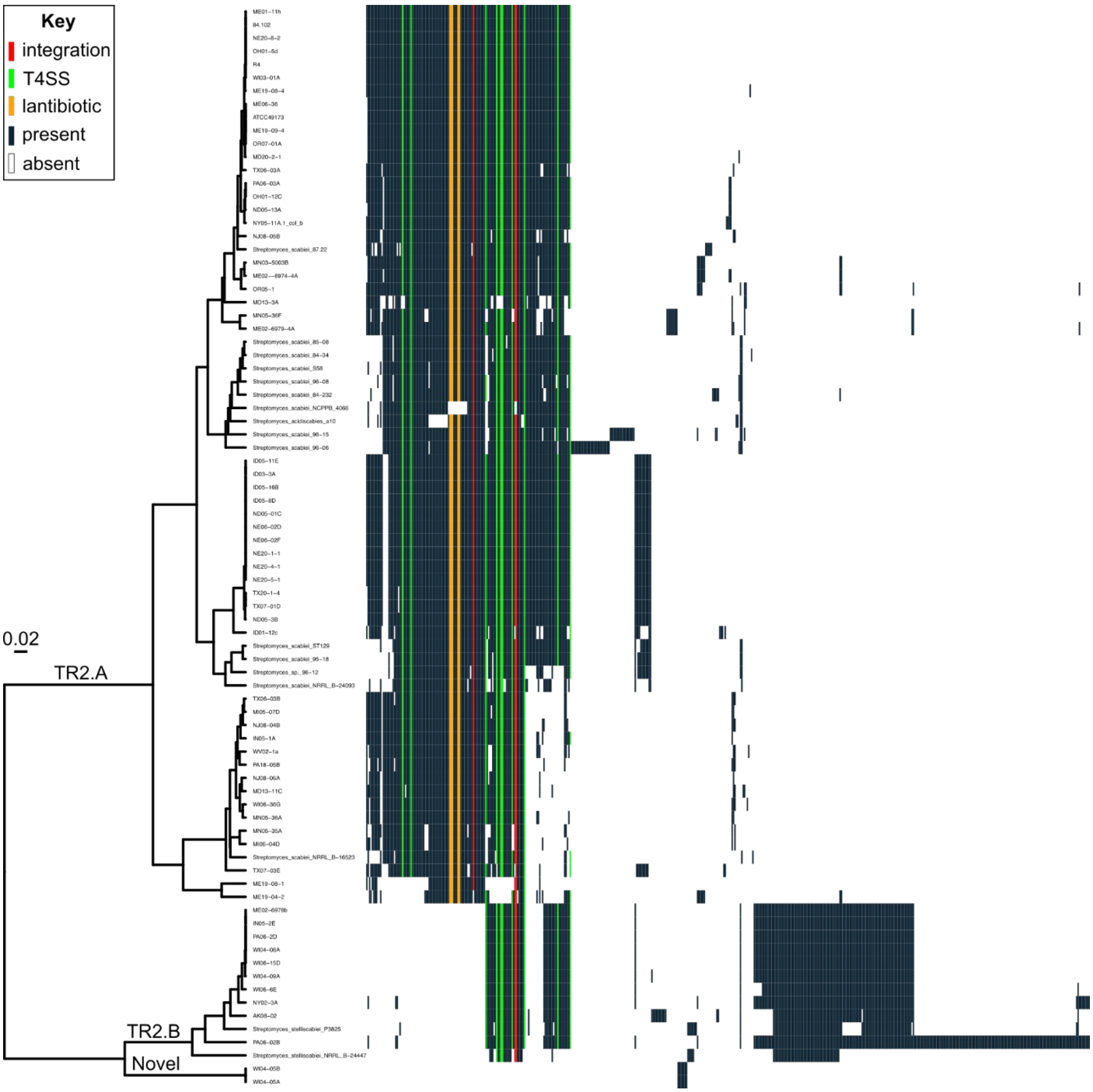

Figure S4. TR2 ICEs form two subtypes based on sequence and gene content. A dendrogram based on row clustering is shown on the left. Columns show presence (black box) and absence (white box) of homologs in TR2 and the novel TR2-like element. Genes with certain functions are highlighted in color. Hierarchical clustering was based on ward.D2 clustering of binary distances. A dendrogram based on column clustering is not shown.

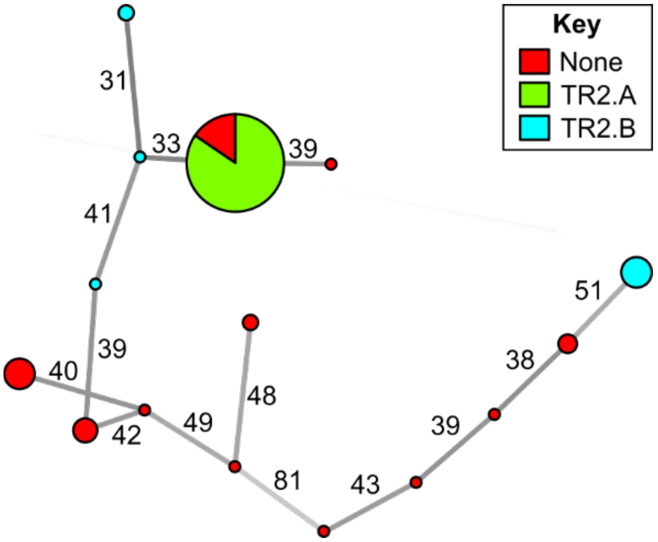

Figure S5. TR1.A is associated with one SNP variant of TR2A. Minimum spanning network of TR1.A IMEs. Nodes representing SNP variants of TR1.A are scaled according to the number of variants (smallest is 1 member; largest is 82 members). Colors in nodes represent the sub-type of TR2 adjacent to TR1.A. Edges linking nodes are labelled with the number of pairwise SNP differences between types. Darker colored edges indicate fewer SNP differences.

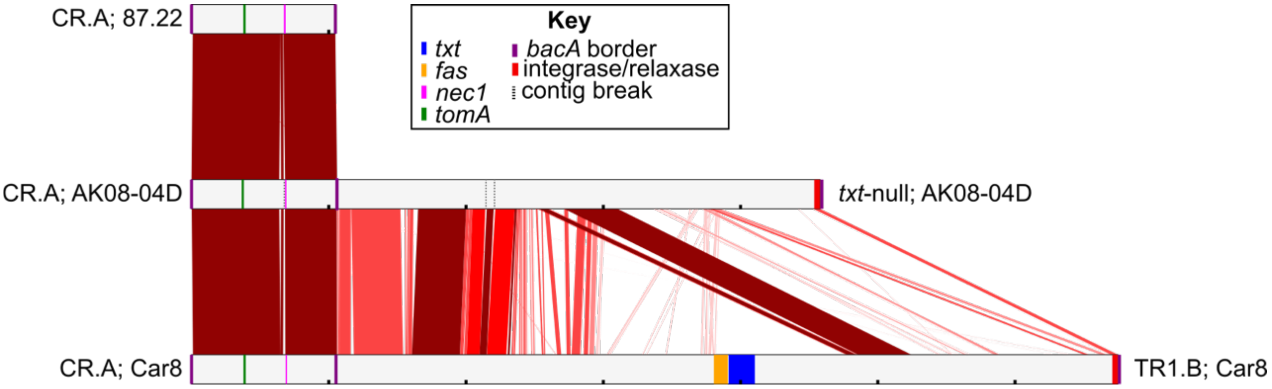

Figure S6. A novel variant of the *bacA* island lacks *txt* genes. Sub-types of MGEs from representative strains are depicted as gray bars with key virulence loci colored. Similarities between MGEs are indicated by red bars, darker colors indicate greater similarity.

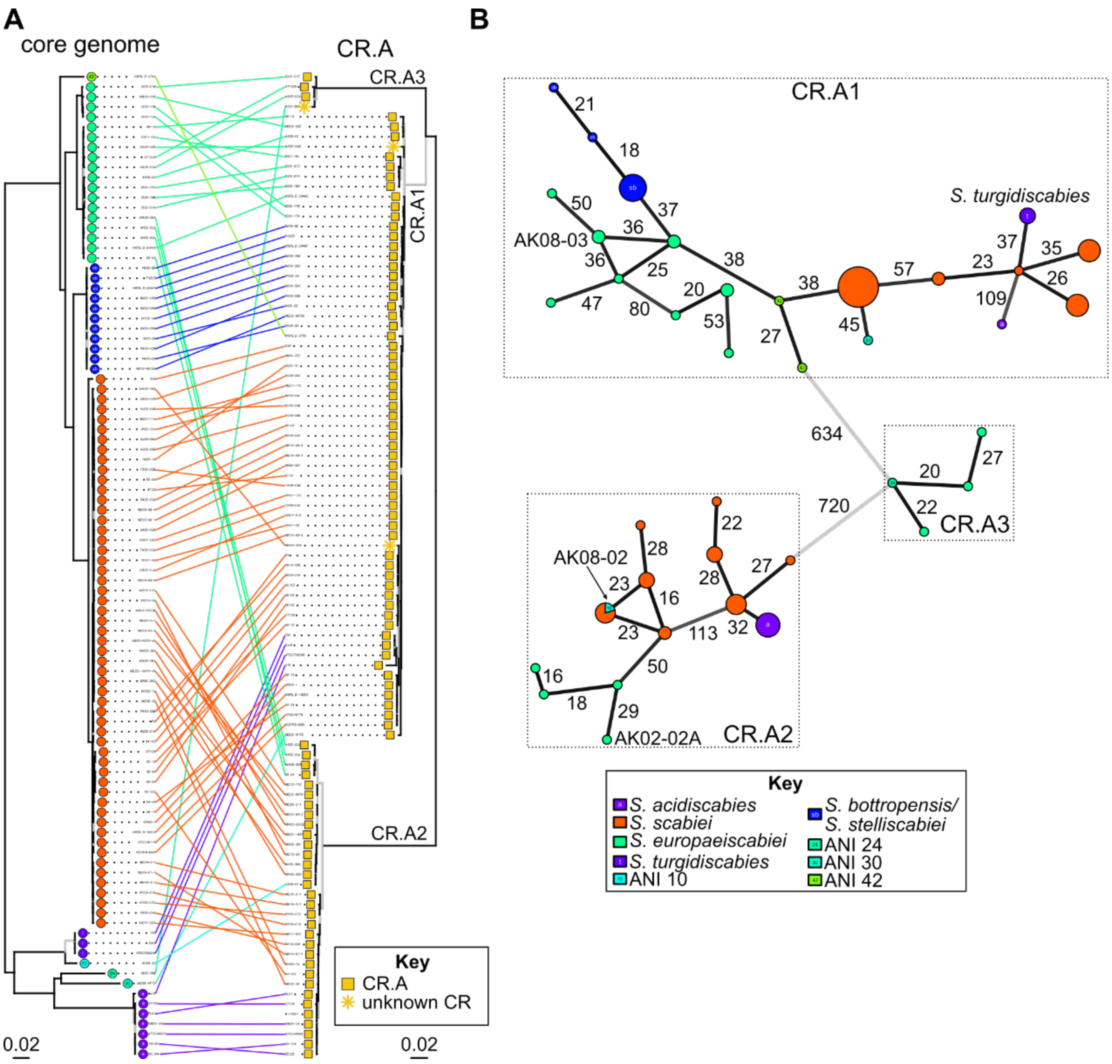

Figure S7. CR.A was horizontally transferred among *Streptomyces* strains. A) Co-phylogeny plot comparing the evolutionary history of strains (left; core genome tree) and CR.A SNP variants (right). Lines connect strains to their corresponding CR.A SNP variant. Tips of the core genome tree are colored according to ANI or species group. The trees are midpoint rooted. Branches with UFBoot > 95% and SH-aLRT > 80% are colored black, otherwise colored gray. **B)** Minimum spanning network of CR.A. Nodes representing SNP variants of CR.A are scaled according to the number of variants (smallest is 1 member; largest is 20 members). Colors in nodes represent the species or ANI group designation of the strains associated with the SNP variant. Edges linking nodes are labelled with the number of pairwise SNP differences between types. Darker colored edges indicate fewer SNP differences.

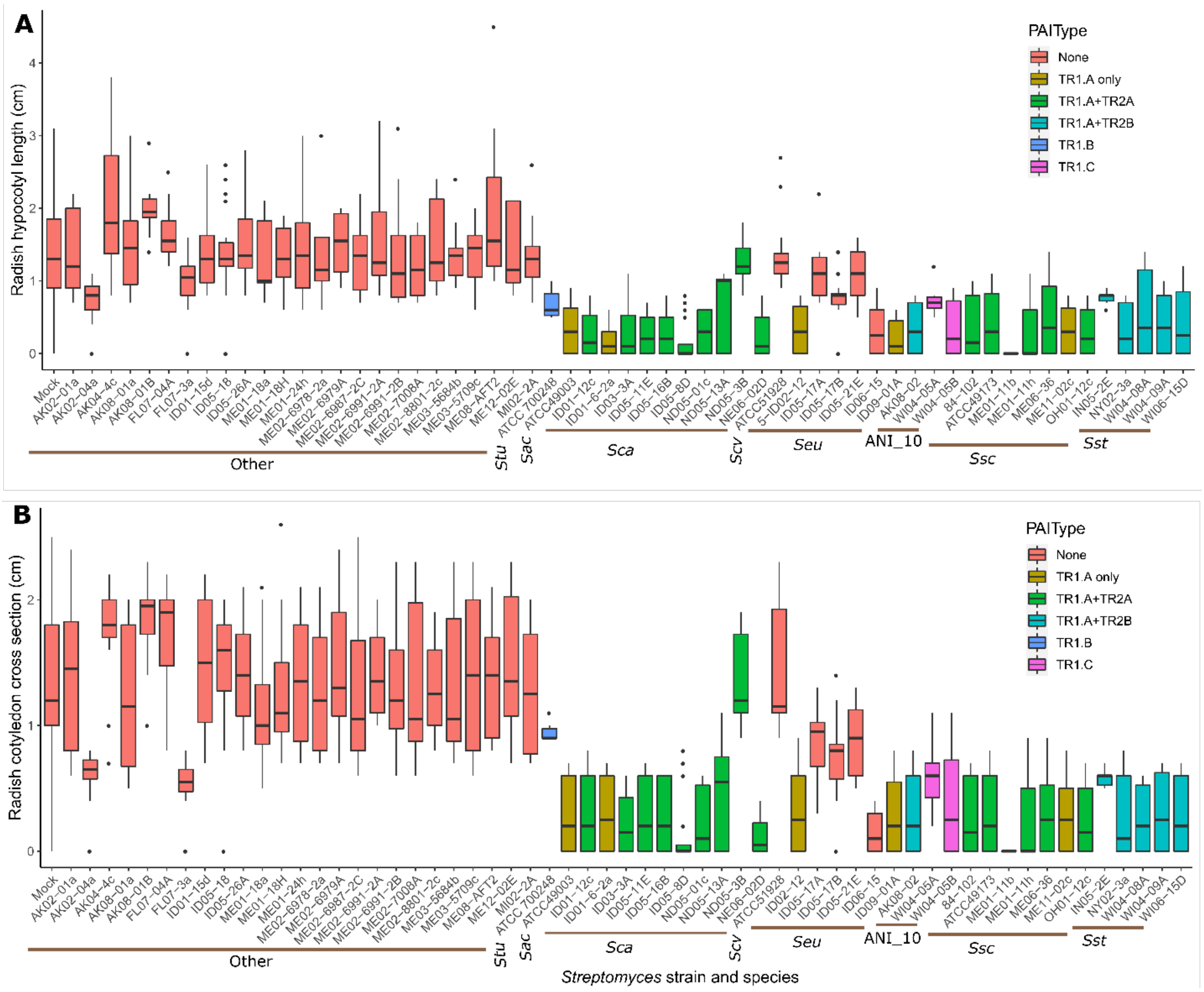

Figure S8. Inoculation of *txt*-carrying strains onto radish attenuates cotyledon expansion and root elongation. A) Stunting of radish seedlings, as measured by hypocotyl length following inoculation with mycelial mass of indicated *Streptomyces* strains for nine days. n = 12 individual plants for each strain. **B)** Cotyledon cross section measurements of the same plants quantified in panel A. Species abbreviations: *Sac, S. acidiscabies; Sca, S. caniscabiei; Scv, S. caviscabies; Seu, S. europaeiscabiei; Ssc, S. scabiei; Sst, S. stelliscabiei*.

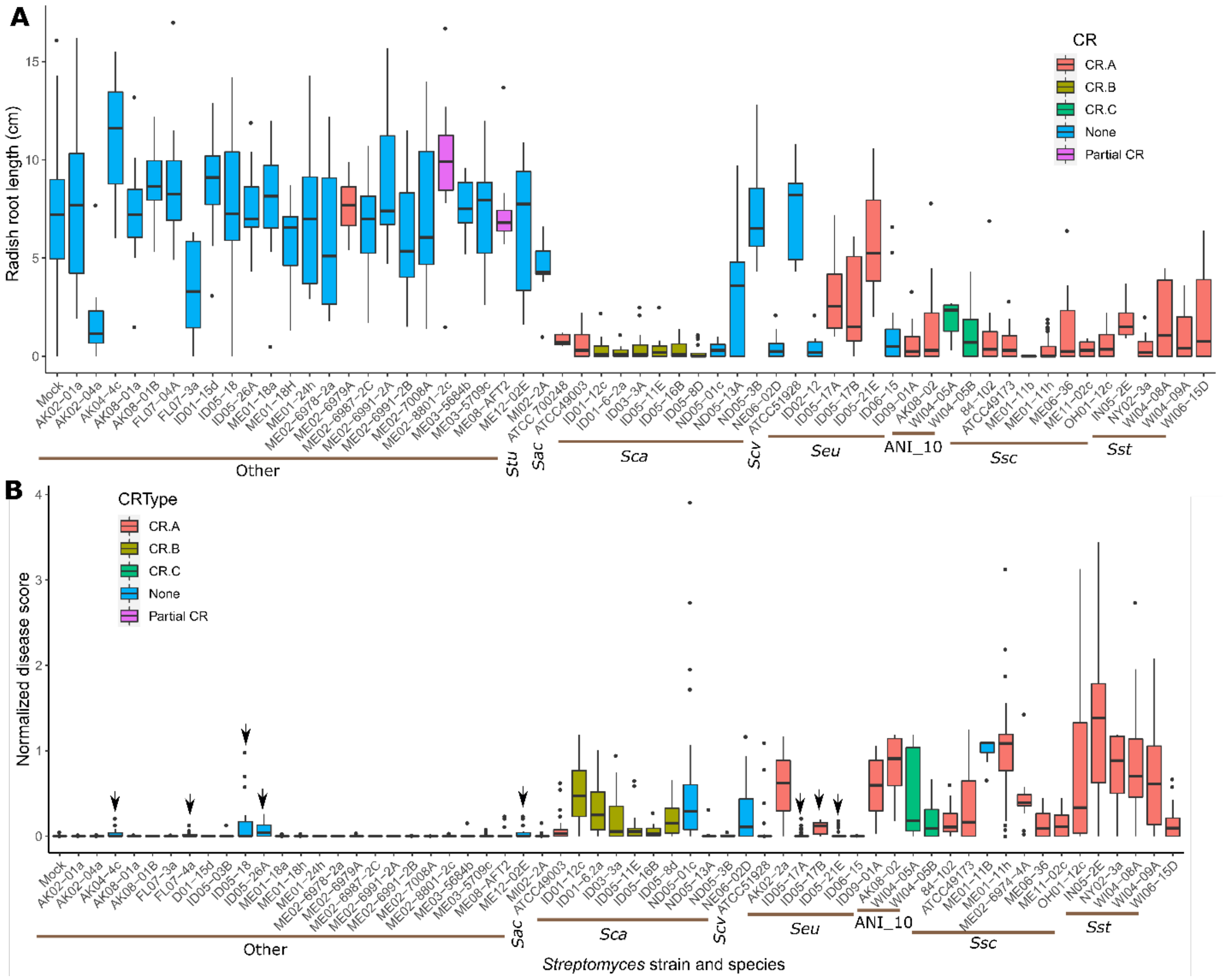

Figure S9. CR subtype does not explain the diversity of plant virulence caused by *Streptomyces* strains. **A)** Stunting of radish seedlings, as measured by root length, following inoculation with mycelial mass of indicated *Streptomyces* strains for nine days. n = 12 individual plants for each strain. **B)** Normalized disease severity on potato cv. Chipewa tubers following inoculation with indicated *Streptomyces* strains in greenhouse-grown pots. All tubers were scored from three replicate pots twelve weeks after planting and inoculation. Data is the same data presented in Figure 5 but color coded to display the CR type. Arrows indicate strains that caused superficial lesions only (indicative of netted, not common, scab) in more than one replicate pot. Species abbreviations: *Sac, S. acidiscabies; Sca, S. caniscabiei; Scv, S. caviscabies; Seu, S. europaeiscabiei; Ssc, S. scabiei; Sst, S. stelliscabiei*.
